# Use of Branched EVAR in Treatment of Juxtarenal Aortic Aneurysm and Essential Accessory Renal Artery: Another Tool on the Shelf? A Case Report

**DOI:** 10.1177/15385744241290011

**Published:** 2024-10-04

**Authors:** Donatas Opulskis, Imam T. P. Ritonga, Philipp Franke, Martin J. Austermann, Marco Virgilio Usai

**Affiliations:** 1 Department of Vascular surgery, 39612 St. Franziskus Hospital, Münster, Germany; 2 Faculty of Medicine, 230647 Lithuanian University of Health Sciences, Kaunas, Lithuania

**Keywords:** juxtarenal aneurysm, accessory renal artery, endovascular surgery, branched endovascular aortic repair

## Abstract

**Objective:**

We present the case of a 58-year-old male patient referred to our department from a smaller facility for further evaluation and treatment strategy regarding the choice between open or endovascular surgery. The patient was diagnosed with a 6 cm asymptomatic juxtarenal aortic aneurysm and a 5 mm diameter accessory renal artery (ARA) supplying the lower half of left kidney. Further diagnostic assessments indicated that the left ARA was perfusing over 40% of the left kidney.

**Methods:**

Given the patient’s significant pre-existing medical conditions and elevated perioperative risk, the decision was made to proceed with minimally invasive endovascular surgery using a custom-made 5-branches stent graft (BEVAR).

**Results:**

In the early postoperative period, the patient reported left flank pain. A subsequent CT scan identified a partial infarction in the left kidney due to the occlusion of an early small branch from the upper left renal artery. However, laboratory results showed no significant change in renal function compared to preoperative values. The patient was discharged 6 days post-surgery, with no additional complications observed during the early postoperative period.

**Conclusion:**

This case report demonstrates that BEVAR is acceptable technique with satisfactory early postoperative outcomes for treating juxtarenal aortic aneurysms with an accessory renal artery in patients who are high-risk candidates for open repair and anatomically unsuitable for FEVAR or Ch-EVAR procedures.

## Introduction

Endovascular aortic repair (EVAR) is commonly utilized to treat juxtarenal aortic aneurysms, yielding favorable early and midterm outcomes.^1^ The choice to proceed with endovascular surgery is primarily influenced by the patient’s comorbidities and age. However, the presence of an accessory renal artery (ARA), present in up to one-third of patients, can complicate the suitability of endovascular treatment and frequently necessitates traditional open surgical repair.^2,3^

## Case Report

**Figure 1.**
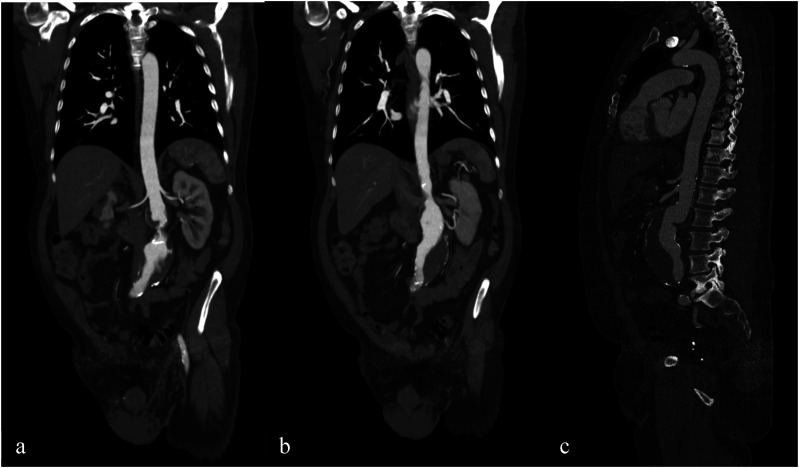
6 cm juxtarenal aortic aneurysm with accessory renal artery on the left kidney originating from aneurysmal sac.

A 58-year-old male patient was referred to our department, from a smaller facility for further evaluation and treatment options regarding the choice between open or endovascular surgery for an asymptomatic juxtarenal aortic aneurysm. The patient’s medical history includes coronary artery disease (CAD), anterior and posterior wall myocardial infarctions subsequently managed with percutaneous coronary intervention (PCI) and stenting, hypertension, chronic kidney disease (Stage I-II), Stage II lung sarcoidosis, hypercholesterolemia, obstructive sleep apnea syndrome, lumbar spine syndrome, a history of paralysis in 1986, and right hip replacement surgery. His current medications include acetylsalicylic acid (ASS), Levothyroxine, Ramipril, Bisoprolol, Rosuvastatin, Ezetimibe, and Agomelatine. Physical examination showed no abnormalities, and the ankle-brachial index was measured at greater than 1. A duplex ultrasound revealed a juxtarenal abdominal aneurysm with a diameter exceeding 6 cm. A subsequent computed tomography angiogram (CTA) (Figure 1) confirmed the presence of a 6 cm juxtarenal aortic aneurysm and a 5 mm accessory renal artery (ARA) on the left kidney. Further diagnostic evaluations indicated that the left ARA was responsible for supplying more than 40% of the perfusion to the left kidney.

Due to the patient’s significant pre-existing medical conditions and elevated perioperative risk, open surgical intervention was deemed unsuitable. Instead, a minimally invasive endovascular approach was selected, employing a custom-made 5-branches stent graft (BEVAR) with a shorter proximal component to mitigate the risk of spinal cord ischemia. The procedure, including its benefits and potential risks, was thoroughly discussed with the patient, and written consent was obtained.

The surgery was performed under general anesthesia with the patient positioned supine. Empirical antibiotic prophylaxis consisting of 1.5 g of cefuroxime and 500 mg of metronidazole was administered prior to the procedure. Three access sites were utilized: bilateral common femoral arteries (CFAs) and the left brachial artery.

The left axillary artery was accessed via a small longitudinal incision, with meticulous layer-by-layer dissection. A Prolene 5-0 U-suture was placed prior to puncture. Following puncture, a 5-Fr sheath was introduced using the Seldinger technique. After administering 5000 IU of heparin, a pigtail catheter was advanced over a stiff wire into the visceral aorta.

**Figure 2.**
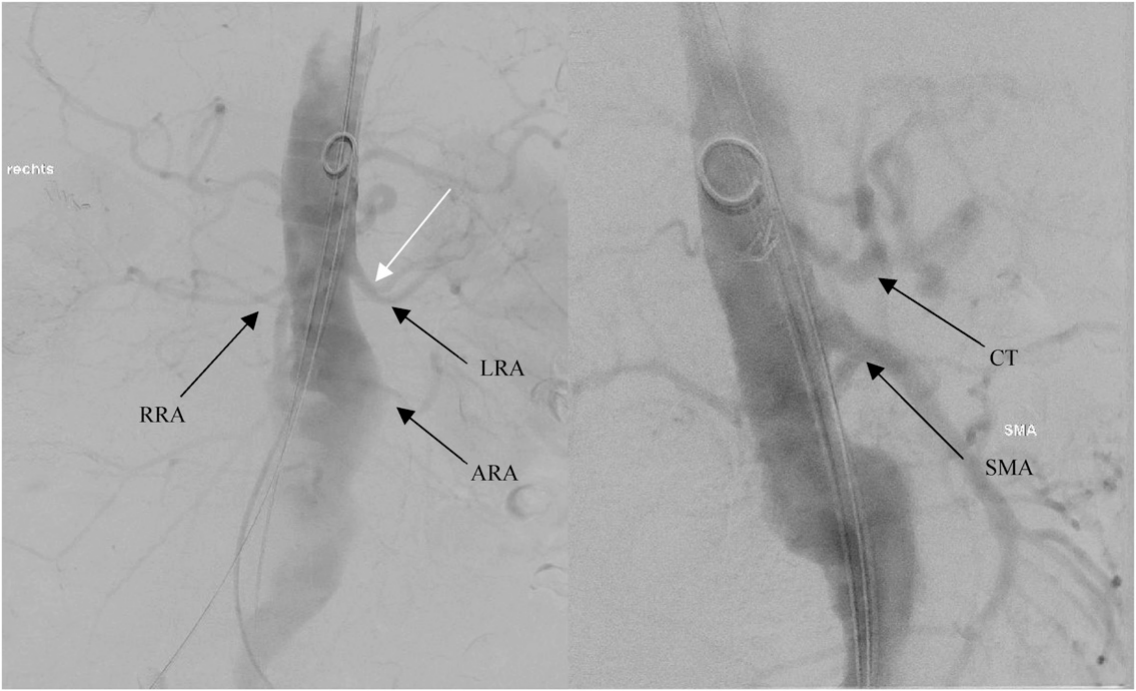
Angiography for a 2D/3D fusion of target vessels prior to stent implantation. All visceral arteries were patient throughout their length.

The CFAs were then accessed through small groin incisions, and after digital blunt preoparation, the anterior walls of the arteries were exposed. Using the Seldinger technique, 8-Fr sheaths were introduced. After placing a Prostar-XL device (Abbott Vascular, Santa Clara, CA, USA), a 14-Fr sheaths were then used bilaterally. A protection catheter with a Lunderquist wire was advanced through the right CFA to the level of the aortic valve, while a pigtail catheter was inserted through the left groin and positioned at the L1 segment. Preliminary angiography (Figure 2) was conducted for 2D/3D fusion imaging of the target vessels before proceeding with stent implantation.

**Figure 3.**
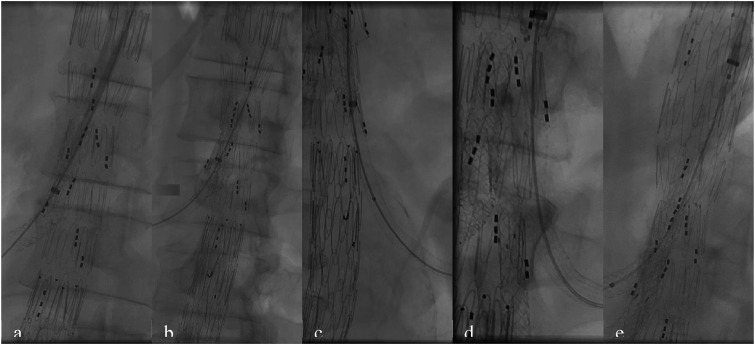
Catheterization and stenting of visceral arteries respectively: SMA (A) two 8 mm BE were used, right renal artery (RRA) (B) 6 mm BE and SE were used, left accessory renal artery (LARA) (C) 6 mm BE and SE were used, left renal artery (LRA) (D) 6 mm SE was used, truncus coeliacus (TC) (E) 8 mm BE was used.

The main body of the 5-branches CMD stent graft was introduced through the right groin via a 22-Fr sheath and positioned approximately 1.5 to 2 cm above the target vessels. It was then deployed, ensuring that all branches were properly aligned and accessible. A bifurcated graft was subsequently advanced and deployed, with the contralateral leg positioned 1-2 cm above the bifurcation. Following deployment, the stent graft for iliac arteries were implanted, and balloon molding was performed to ensure proper graft expansion and adherence. Both CFA were closed using percutaneous closure devices, and pulsations were verified in both groins. Subsequently, access through the right brachial artery, where 12-Fr and 8-Fr sheaths had been introduced, allowed for catheterization and stenting of the visceral arteries (Figure 3).

**Figure 4.**
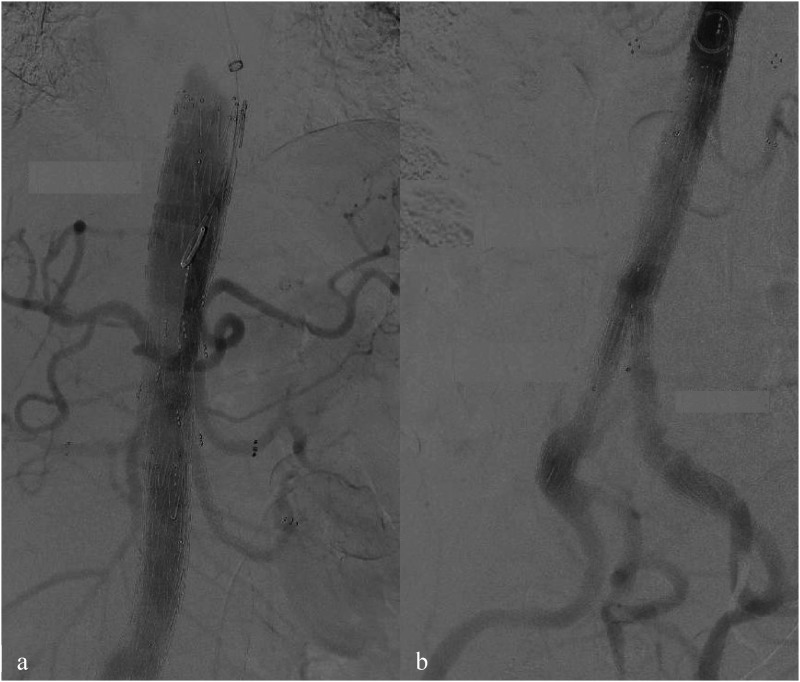
Final angiography after 5 branch CMD implantation (A) with bifurcated graft and iliac legs extensions (B). All visceral arteries are patient with no signs of endoleaks or dissection observed.

During the LRA stenting, one branch was intentionally covered to ensure a proper distal seal (**white arrow,** Figure 2). Once all target vessels were stented, a final angiographic assessment was performed to identify any signs of dissection or endoleaks (Figure 4). Thereafter, the brachial artery access site was closed. A drainage tube was inserted, and the incision was meticulously closed in layers using a continuous suturing technique.

Following the procedure, the patient was extubated in the OR and transferred to the ICU for 48 hours of observation. In the early postoperative period, the patient reported pain in the left flank. A CT scan revealed a partial infarction of the left kidney due to the coverage of a branch from the left renal artery. Despite this finding, laboratory results showed no significant changes in renal function compared to preoperative levels. The patient was discharged 6 days post-surgery with no additional complications noted during the early postoperative period. The patient was advised to continue dual-antiplatelet therapy as part of their postoperative management.

## Discussion

This case report presents an atypical but effective treatment option available for patients diagnosed with asymptomatic juxtarenal aortic aneurysm who also have an ARA with a high percentage of kidney perfusion. Current published data is inhomogeneous regarding the treatment of ARA. Some authors advocate for embolization and coverage of the ARA, arguing that the occlusion of accessory renal arteries is not associated with clinically significant symptoms, even in patients with renal insufficiency.^4^ Others contend that if the ARA is greater than 4 mm and contributes more than 30% to kidney perfusion, it should be preserved to prevent renal function decline.^5,6^

In our case, the patient had already been diagnosed with stage 1-2 renal insufficiency, and the ARA supplied over 40% of the left kidney. Therefore, the decision was made to preserve the ARA. While open surgical repair remains the gold standard for juxtarenal aortic aneurysms, it should only be considered for patients without significant comorbidities affecting cardiac, pulmonary, or kidney function. This approach is associated with a higher early postoperative mortality rate due to complications such as multiorgan failure, myocardial infarction, mesenteric ischemia, and renal impairment, which can result from cross - clamping the aorta above the renal arteries.^7^ Given these risks, it was decided to avoid open surgery.

Endovascular treatment options are described as an alternative to patients who are not suitable for an open repair. Several methods are described in the literature, including FEVAR, Ch-EVAR, periscopes, snorkels, and physician-modified endografts (PME). There is ongoing debate about which strategy is superior. FEVAR is the preferred endovascular treatment option when feasible and it is associated with lower rates of type I and III endoleaks and better target vessel patency compared to other endovascular options.^7,8^ However, it is time dependent due to the need of customization.^9^ In our case, FEVAR was not approved by the customizing company because of the proximity of the ARA, SMA and LRA, which made this approach unfavorable.

Ch-EVAR might be another alternative, known for being cost-effective and accessible, but it carries a higher risk of endoleaks, especially type IA due to gutter formation, as well as increased rates of occlusion and reintervention.^9-11^ Because of the young age of the patient and the necessity of a 4-fold Ch-EVAR, this treatment option was not considered. Physician-modified endografts (PME) are another option, with some studies suggesting their use as a feasible alternative for the treatment of juxtarenal aortic aneurysm with ARA,^10,12^ demonstrating acceptable mid- and long-term results.^13,14^ However, Ch-EVAR and PME are more commonly used in emergency situations where off-the-shelf and custom-made devices are not available.

Considering all treatment options, the decision was made to perform a BEVAR procedure, which, in this case, demonstrates its viability as an alternative when other treatments are unsuitable. Surely, a certain risk of spinal cord ischemia had to be taken in consideration as it is described in a literature.^15^ In this case the difference in length of aortic coverage compared with a planned fenestrated device was less than 1 cm. Nonetheless, the short proximal sealing was chosen to reduce this risk. However, further studies are necessary to achieve optimal treatment outcomes.

## Conclusion

BEVAR showed acceptable early postoperative results for the treatment of juxtarenal aortic aneurysms with an accessory renal artery in patients who are not good candidates for open repair, and for whom FEVAR or Ch-EVAR procedure is not suitable due to anatomical reasons. Additionally, the risk of spinal cord ischemia could be mitigated with reduction of aortic coverage through customization.

## Appendix

### Abbreviations

ARA: Accessory renal artery
BEVAR: Branched endovascular aortic repair
CT: Computed tomography
FEVAR: Fenestrated endovascular aortic repair
Ch-EVAR: Chimney endovascular aortic repair
CAD: Coronary artery disease
PTCA: Percutaneous transluminal coronary angioplasty
CTA: Computed tomography angiography
CFA: Common femoral artery
IU: International units
CMD: Costume made device
SMA: Superior mesenteric artery
BE: Balloon expandable stent
SE: Self expanding stent
RRA: Right renal artery
LARA: Left accessory renal artery
LRA: Left renal artery
TC: Truncus coeliacus
ICU: Intensive care unit
